# Pro-Environmental Behaviors and Well-Being in Adolescence: The Mediating Role of Place Attachment

**DOI:** 10.3390/ijerph20105759

**Published:** 2023-05-09

**Authors:** Maria Giuseppina Bartolo, Rocco Servidio, Anna Lisa Palermiti, Maria Rosaria Nappa, Angela Costabile

**Affiliations:** 1 Department of Culture, Education and Society, University of Calabria, 87036 Arcavacata di Rende, Italy; rocco.servidio@unical.it (R.S.); annalisa.palermiti@unical.it (A.L.P.); a.costabile@unical.it (A.C.); 2 Department of Systems Medicine, Tor Vergata University of Rome, 00133 Rome, Italy; maria.rosaria.nappa@uniroma2.it

**Keywords:** pro-environmental behaviors, well-being, place attachment, adolescence

## Abstract

Adolescents represent the future generation, so it is important to pay attention to behaviors that involve them as actors in social activities and constitute the expression of an adequate growth path. Engaging in pro-environmental behaviors leads adolescents to do something good for themselves, for their own community, and for the place in which they live, and this type of conduct increases their levels of well-being and place attachment. This study examines the association between pro-environmental behavior and personal and social well-being in a sample of 1925 adolescents aged 14 to 20 years. Structural equation analyses showed a direct positive effect of pro-environmental behavior on personal and social well-being as well as place attachment. The latter partially mediated the relationship between pro-environmental behaviors and personal and social well-being. This study is significant in that it provides new data on how pro-environmental behaviors enhance adolescents’ personal and social well-being by potentially ensuring long-term benefits, thereby suggesting that it is important to stimulate, motivate, and recommend these kinds of actions.

## 1. Introduction

It is widely known that adolescence is a sensitive period for shaping behaviors, as it provides opportunities to influence developmental trajectories [1], face the various developmental tasks required by society, make independent choices also regarding one’s future [2], engage in a wider variety of prosocial actions [3], and promote prosocial behavior [4].

According to the theoretical perspective of PYD, adolescents’ involvement in social activism, or in initiatives to promote social change, is the expression of an adequate growth path; thus, it is important to investigate the relationships between individuals in connection with their development in their ecological context [5] on the basis of the actions they perform. As a dynamic part of their community, and as current and future agents of change [5,6,7], adolescents can act in such a way as to enhance the place in which they live and, consequently, the world by engaging in pro-environmental behaviors. This expression refers to a kind of concern for oneself, others, and the ecosystem more generally that leads people to adopt behaviors useful for promoting environmental sustainability [8].

Although all individuals in their lifetime can contribute toward environmental preservation and protection through responsible and eco-friendly behaviors, different studies [9,10,11] have underlined that youth tend to be more concerned about the environment and more aware of the possible damage resulting from environment neglect, and hence more likely to engage in pro-environmental behavior. Indeed, adolescents pay particular attention to the environment as a dimension that may negatively affect their own well-being, physical and mental health, and personal and interpersonal development [7,12].

Various studies have shown that pro-environmental behaviors are positively associated with people’s subjective well-being [13,14]. Recently, Ramkissoon and colleagues [8,15] have demonstrated the positive impact of pro-environmental behaviors on people’s lives and hence on well-being levels, highlighting the fact that pro-environmental behaviors can also bring high place attachment and improve one’s quality of life.

The promotion of healthy behaviors and engagement in activities related to the safeguarding of the natural environment have important benefits for people [16,17,18], considering that pollution, energy expenditure, and fossil fuels have a significant impact on the environment and health, with negative consequences on well-being. However, to our knowledge, there are no previous studies examining the importance of pro-environmental behavior for both personal and social well-being.

Once a positive behavior is adopted, its positive effects tend to reinforce the tendency to repeat the same behavior over time. This leads people to maintain behaviors that in the long term promote a sense of belonging and attachment to one’s place, which thus undergoes continuous improvement [19,20,21].

On the basis of such premises, the current study tests a model that considers the effects of adolescents’ pro-environmental behaviors on personal and social well-being through the mediating role of place attachment. The added value of this model is represented by the opportunity it offers society and all educational settings to devise and promote intervention programs based on the diffusion and acquisition of knowledge about the importance of applying pro-environmental behaviors [18] so as to foster social changes that are likely to enhance personal and social well-being. Promoting pro-environmental behaviors among adolescents could have a direct positive impact on their well-being and could also positively influence their place attachment, since place attachment is fundamental to their well-being (it provides pleasure and comfort that help individuals feel good about themselves and others) and is also developed through actions carried out in and for the place itself.

### 1.1. Adolescence and Pro-Environmental Behaviors

During adolescence, youth searching for their own identity and place in society experiment with different roles, as they begin to perceive themselves as members of a community where they can play an active part [22,23] by taking part in collective or independent actions, such as behavior that protects the environment and improves its sustainability.

Since the mid-1960s, different disciplines, such as geography, environmental planning and design, natural resources management, sociology, and psychology, have shown an interest in environmental behaviors [24]. Especially psychology has allowed us to better understand the complex interplay between the environment, individual development and well-being through combined developmental and bioecological perspectives [25]. Today, the most widely accepted definition of pro-environmental behavior identifies it as all actions that can reduce the negative impact of human life on the environment [26,27]. These behaviors are exemplified by actions such as recycling [26,27,28,29,30], water-saving (having shorter showers, closing the tap when brushing one’s teeth or after washing one’s hands), energy waste reduction [31], waste management [32,33,34], and food waste avoidance [35,36].

In recent decades, owing to the major climate changes we are facing, increasing attention has been paid to the environment and its resources, which are being depleted at increasing rates. Many pro-environmental organizations have been established to spread knowledge and information about the correct behaviors to be adopted to protect the world. In particular, many studies have underlined that among youth, there has been an increase in behaviors such as recycling, buying green products, and avoiding over-consumption [11,37,38]. Academic research on pro-environmental behavior is growing steadily, as no general consensus about its theoretical bases has yet been reached [39]. While previous studies on pro-environmental behaviors have been based on adult samples, it is important to investigate pro-environmental behaviors among adolescents, as adolescence is the phase of life in which norms and values are formed and/or consolidated, defining individuals’ personal and social identity.

In Europe, we have witnessed a real media sensation: Greta Thunberg, a young Swedish activist who has led demonstrations against climate change. Many other teenagers from all over the world have taken part in her initiative “Fridays for Future” [40]. The fact that such protests take place outside the family context might be a motivating factor for young people because it encompasses social interaction with their peer group. Adolescence is a time of life when social interactions and identifying with one’s peers outside the family are particularly important. In this phase of life, personal and social identities develop, along with moral and emotional aspects, through the adoption of high-value behaviors. For instance, pro-environmental behaviors may be driven by an internal motivator (e.g., self-identity) or by perceived norms [41,42] with reference to one’s membership group.

Few studies [43,44] have considered the role of the environment in the framework of positive youth development (PYD). Pro-environmental behaviors lead adolescents to do something good for themselves and for their own community, and behaviors of this sort increase adolescents’ level of life satisfaction [45] and, consequently, of well-being.

Even if engaging in pro-environmental behaviors does not always have immediate benefits for the person involved, sometimes the benefits are only long-term ones. Doing the “right” thing, perceiving one’s actions as good, will make the individual feel that he/she is a good person; consequently, it will make him/her feel good [46,47].

### 1.2. Well-Being in Adolescence

Well-being is defined as global life satisfaction [48] and it essentially takes two forms: personal psychological well-being and social well-being. Personal and social well-being are two conditions in adolescents’ lives that are important insofar as personal life satisfaction and positive relationships are crucial for teens when they are building their identity [49].

Personal psychological well-being is a subjective condition of life satisfaction: it is identified with positive mental health [50], which includes different aspects, such as affectivity [51,52], self-life-satisfaction, happiness, and positive relationships [49]. It can be defined as a condition in which positive feelings outweigh negative ones, and a state of subjective well-being that is not only individual but also positively influences social relations and social participation [53].

Social well-being regards the assessment of one’s condition in relation to one’s role in society [23]. Social well-being is also the result of an individuals’ contribution as a member of society, i.e., of what they do in the world and how they contribute to the common good; it thus reflects communal experiences based on social goals. Social well-being also regards the extent to which a person feels part of a social group, of the community where he/she lives, and the way in which he/she actively contributes to the pursuit of a common goal with other people; consequently, it also refers to positive attitudes toward others. Social well-being is very important during adolescence, as it reflects the degree to which youth are willing to be active in their community so as to enhance it and contribute to society [54].

### 1.3. Place Attachment in Adolescence

Place attachment also contributes to adolescents’ psychological well-being [15,55].

Place attachment can be considered a multidimensional construct [56,57,58] that originates from attachment theory [59] and reflects the emotional and social ties binding people to specific settings [58,60,61]. People tend to become attached to the place from which they receive satisfaction, benefits, and comfort [15,55,62,63] and which helps them feel good and reach high levels of well-being. However, this attachment also stems from the fact of experiencing a place on a daily basis: it is also formed through the *practicing of place* [64,65]. People become attached to a place not only by establishing emotional and social ties with it, but also by carrying out actions that help them to feel good and part of that particular place.

Since adolescents find themselves in a phase of identity maturation, they transform what they have inherited from their parents by adapting it to their own context [66]. They also do so through new behaviors, ideals, values, and beliefs that meet their needs. This means that adolescents who implement certain behaviors (e.g., pro-environmental behaviors) create significance for a particular place and participate in place-making that affects their knowledge and thus their individual place attachment. The meanings that adolescents assign to places inform their emotional and practical responses to such places as adults, which is why when adults are asked about their favorite place, they often describe the one they contributed to creating during their childhood [67].

People feel place attachment because in their daily lives they implement actions to improve the place where they live. In light of Hidalgo and Hernández’s [58] suggestion that attachment should be explored in relation to different places, we aimed to assess whether pro-environmental behavior can foster attachment to a large place (a city).

The relationship between pro-environmental behaviors, place attachment, and well-being [63] certainly reflects a strong link between places and people, so the attachment bond might be a key to explaining the relationship between pro-environmental behaviors and well-being, on both the personal and social level. It is important to know people’s relationship with the place where they live in order to better understand their quality of life and, consequently, their level of personal and social well-being [68].

In light of this, we can assume that place attachment mediates the relation between pro-environmental behaviors and well-being.

Our thinking in the current study is that, over time, pro-environmental behaviors help adolescents to improve their levels of personal and social well-being. This hypothesis seems plausible, considering that the environment is one of the most important components for humankind and that all norms and values that drive people to adopt high-value behaviors, such as pro-environmental ones, will promote good environmental conditions, which in turn will have a positive impact on human life quality [69]. As part of the community, adolescents can enact pro-environmental behaviors that directly or indirectly influence their future [70] and well-being.

If we accept that place attachment does not only have to do with the psychological concept of rootedness, but is also the result of the material actions that we carry out in a particular place, it also seems reasonable to assume that taking care of the environment, through pro-environment behaviors, promotes place attachment, as it involves emotions, knowledge, beliefs, and positive behaviors with regard to a place [71,72]. Since the place where people live provides satisfaction, benefits, and comfort that help them to feel good, the present research hypothesizes that place attachment plays a mediating role between pro-environmental behavior, on one hand, and personal and social well-being, on the other.

Through a mediation model, the current research offers a contribution to the literature, first by underlining the effectiveness in terms of well-being of applying pro-environmental behavior in adolescents’ life and, second, by identifying the mediating role of place attachment as a factor contributing to a healthy life.

## 2. Materials and Methods

### 2.1. Participants and Procedures

The research was carried out through an online questionnaire between April and May 2021. All state high schools in the Calabria region were invited to participate in the survey. Those schools that agreed to participate recruited participants through their directors and then through teachers, who invited students to take part in the research. Participation in the study was voluntary and anonymous. A total of 1925 adolescents (M = 908 (47.2%), F = 1017 (52.8%)) aged 14–20 years (Mage = 16.3 DS = 1.46) agreed to take part. As most of the students were minors, the schools obtained parental consent. All information about the nature and purpose of the study was disseminated by teachers and was written at the top of the first page of the online survey, along with information about the anonymity of the research. An e-mail address was provided at the end of the questionnaire for any questions and doubts about the survey. The average time spent to complete the online survey was 15–20 min.

The study procedures and materials were designed and employed according to the ethical standards laid out by the Italian Psychological Association (AIP).

### 2.2. Measures

The online questionnaire consisted of a battery of socio-demographic profiles and self-report scales.

*Socio-demographic profile*

Participants were asked to report general information about their gender, age, parental education and employment, and current place of living.

*Pro-environmental behaviors*

To assess pro-environmental behaviors, 11 items used in previous research [73,74,75] were selected on the basis of behaviors in which adolescents are likely to engage. Thus, items included energy and water conservation (e.g., “I turn off TV and computer screens when they are not in use”; “I turn off the water faucet when I brush my teeth”), waste recycling (e.g., “When I’m out I worry about throwing waste in the correct bins”), and fuel consumption reduction (e.g., “I use public transportation to get around in my city”). For all items, participants were asked to indicate on a scale Likert how often they engaged in these behaviors (from 1 “never do this” to 5 “always do this”). The reliability value for the present study was α = 0.65.

*Warwick–Edinburgh Mental Well-Being Scale (WEMWBS)*

The Italian version of WEMWBS [76] includes 12 items (e.g., “I have been feeling optimistic about the future”), which are all positively worded in relation to each statement. Respondents are required to describe their experience over the past two weeks using a 5-point Likert-type scale ranging from 1 (never) to 5 (always). A higher WEMWBS score, therefore, indicates a higher level of mental well-being. The reliability value for the present study was α = 0.88.

*Social well-being*

A 5-item short version of the Social Well-being Scale [23] was used to assess the perception of one’s relations with the community and society and the perceived quality of its functioning within it. Each item was measured on a 6-point Likert-type scale from 1 (never) to 6 (every day) (e.g., “How many times have you felt that you had something important to offer to society”). Cronbach’s alpha was 0.81.

*Place attachment*

Place attachment was measured with a 3-item “city” subscale, partly based on Lewicka’s scale [77,78]. Each item was rated on a 5-point Likert-type scale from 1 (strongly disagree) to 5 (strongly agree). The items were: “I like this city”, “I feel attached to this city”, and “I am proud of this city”. The reliability of the scale was α = 0.67.

### 2.3. Statistical Analyses

The statistical analyses, descriptive statistics, and bivariate correlations (Pearson’s r) were performed through SPSS-27. All the variables were sufficiently normally distributed (items had skewness and kurtosis in the +1 to −1 range). We did not have missing data, as we required responses for all items. We computed bivariate correlations (Pearson’s r) among the variables of interest and control variables (age and gender). In addition, the reliabilities of the scales and subscales were estimated by computing Cronbach’s α. To test the study’s hypotheses, a structural equation modeling (SEM) analysis was performed using Mplus, version 7.01 [79]. As the first step in the SEM, the measurement model for the latent constructs was tested. Furthermore, we tested the full (measurement and structural) model. In the last step, a mediation analysis was performed. The models were estimated with the maximum likelihood (MLR), with standard errors and a mean adjusted chi-squared test statistic robust to non-normality. To ascertain the model fit, we used the comparative fit (CFI), Tucker–Lewis (TLI), and root-mean-square error of approximation (RMSEA) indexes. According to Kline [80], we considered values of CFI ≥ 0.95, TLI ≥ 0.95, and RMSEA ≤ 0.05 to be indications of good model fit.

## 3. Results

Table 1 shows the results of the descriptive and the bivariate Pearson’s r correlations. All the associations between the main variables of the study were positive and significant, and this result satisfied the conditions for performing the next analyses. However, regarding the demographic variables (age and gender), we found negative and significant associations with the other constructs, except for pro-environmental behavior.

**Figure 1:**
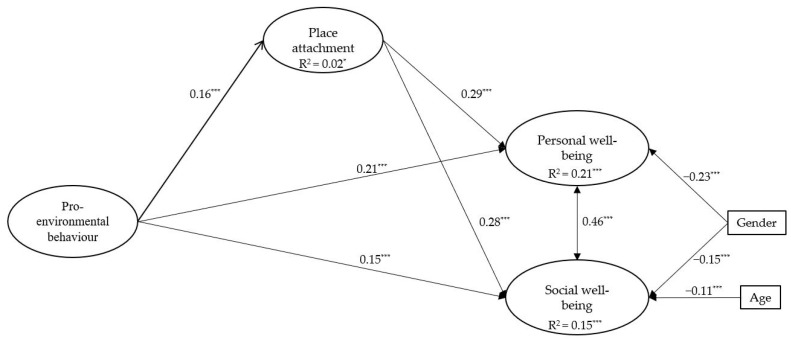
Results of the SEM model. All the values are standardized. Note: * *p* < 0.05. *** *p* < 0.001.

The results of the measurement model including all the variables fit well with the data, with a robust χ^2^(411, N = 1925) = 1284.20, *p* < 0.001, CFI = 0.94, TLI = 0.93, RMSEA = 0.03, 90% CI [0.031, 0.035], and SRMR = 0.041. Since the measurement model results were satisfactory, we modeled the effects among the latent variables to test the study’s hypotheses. The results of the SEM analysis (measurement and structural) are shown in Figure 1. The tested model, which includes gender and age as covariates, fits the data well, and has a robust χ^2^(469, N = 1925) = 1638.22, *p* < 0.001, CFI = 0.92, TLI = 0.91, RMSEA = 0.04, 90% CI [0.034, 0.038], and SRMR = 0.043.

The results shown in Figure 1 suggest that pro-environmental behavior has a direct and positive effect on place attachment, β = 0.16 and *p* < 0.001; personal well-being, β = 0.21 and *p* < 0.001; and social well-being, β = 0.15 and *p* < 0.001. In turn, place attachment positively affects personal well-being, β = 0.29 and *p* < 0.001, and social well-being, β = 0.28 and *p* < 0.001.

Place attachment partially mediated the relationship between pro-environmental behavior and personal well-being, with β = 0.05, SE = 0.01, t = 3.92, *p* < 0.001, of the total effect, β = 0.26, SE = 0.03, t = 7.58, *p* < 0.001; and social well-being with β = 0.04, SE = 0.01, t = 3.78, *p* < 0.001 of the total effect, β = 0.19, SE = 0.03, t = 5.79, *p* < 0.001.

## 4. Discussion

This study explored the relation between pro-environmental behaviors and well-being among adolescents, more specifically by identifying the mediating role of place attachment as a factor contributing to a healthy life.

Our main findings show that pro-environmental behavior has a direct positive effect on place attachment and well-being; in turn, place attachment positively affects well-being. Furthermore, place attachment partially mediated the relationship between pro-environmental behaviors and well-being.

As a form of pro-social behavior, pro-environmental behaviors have an enduring effect on present and future well-being in several ways: for example, adopting high-value behaviors helps adolescents to build their own identity and, consequently, to feel good vis-à-vis the rest of society; thinking of doing something to improve the world promotes the achievement of a more comfortable life in better environmental conditions [81]. Another reason as to why pro-environmental behaviors are likely to increase well-being is that living a more comfortable life in better environmental conditions gives meaning to one’s life, fostering a positive self-image, or providing a social identity [82]. These statements help us to understand that engaging in pro-environmental behaviors makes an individual feel good, as these are considered to be correct behaviors: doing “the right thing” for the environment and other human beings makes people feel good [13,83,84].

Furthermore, our study broadens current knowledge in this area, since it investigated the effect of pro-environmental behaviors on social well-being.

Given that the European Union pays considerable attention to environment problems [85] through campaigns to raise environmental awareness, ads, policies, etc., pro-environmental behaviors could be regarded as socially normative goals with positive moral value [86]. Social well-being consists in the evaluation of one’s own contributions as a member of society: it concerns the relationship between individuals and society and, consequently, the way in which individuals actively contribute to the common good by working with others. Therefore, we can affirm that pro-environmental behaviors have an impact on social well-being. Insofar as they offer an opportunity to experience participation in one’s community, pro-environmental behaviors could add value to the psychological aspect of becoming part of a community. We believe that pro-environmental behaviors, once learned, may be activated in an automatic and habitual way with long-term benefits in terms of well-being.

Although social well-being is influenced by people’s values and goals, it is important to underline that it also depends on life experiences, social relationships, and one’s sense of connectedness with people and places, particularly the meaning one assigns to one’s place of belonging. Therefore, our study also considered the role of place attachment in the relationship between pro-environmental behaviors and well-being. Our findings confirmed an indirect significant association between the two through the mediating role of place attachment. In particular, adolescents who engage in pro-environmental behavior feel more attached to their place and hence have higher levels of well-being.

The results from studies on the role of place attachment are still inconsistent: some studies underline that there is a significant correlation between place attachment and pro-environmental behaviors [21,87,88], others affirm that no such correlation exists [59,89], and others still affirm that place attachment predicts pro-environmental behaviors. Although place attachment may lead people to become aware of threats to the environment and to strive to protect their place of belonging through pro-environmental behaviors, as Ramkissoon and colleagues [15] have underlined, pro-environmental behaviors can also improve one’s quality of life. When people see the actual impact of their behaviors on their surroundings, they perceive their place as more comfortable. Positive new habits will thus foster long-term behavioral changes that, in turn, will lead to improvements to one’s living place, a higher level of place attachment [19], and beneficial effects in terms of well-being. Individuals’ interactions and bonds with a specific place are the results of their commitment, responsibility, and management of the place [90,91] in terms of actions, protection, and conservation [56,77,87,92]. People engaging in these behaviors may become more emotionally attached to their place and this, in turn, may influence their well-being. Moreover, people who feel place-attached develop a stronger sense of community, neighbor relations, and mutual assistance [93,94], which are all elements that reflect social well-being.

Nevertheless, the present results also support the claim that pro-environmental behaviors are indirectly related to adolescents’ well-being through the mediating effect of place attachment. It seems, then, that adolescents’ natural propensity to develop social peer relationships, also by participating in groups, associations, or other forms of organization that are sensitive to environmental issues, is a factor motivating youth to feel that they are playing an active role in society and to do something to improve the place where they live, thereby making them feel more attached to their place. These two conditions in turn foster well-being.

## 5. Conclusions

Environmental problems are among the most topical issues of social, political, and psychological interest. With this study, we intend to contribute to the research highlighting the psychological aspects of the effects of pro-environmental behaviors in adolescents’ lives.

Analyzing an Italian sample, we found that pro-environmental behaviors have an impact on personal and social well-being during adolescence.

In light of this, in order for pro-environmental behaviors to increase well-being, it is important to help people understand that voluntary behaviors of this kind are correct and meaningful [95]; therefore, a far-sighted educational approach is required to effectively stimulate consistent behavior toward the environment. Stimulating, motivating, and recommending action in favor of the environment among adolescents through eco-friendly and pro-environmental behavior can help them create meaning and shared values through collective actions, leading them to attain social and personal psychological well-being [17,18]. Moreover, educational systems, in addition to daily actions (e.g., knowledge, awareness, and actions) [96,97], could improve some students’ capabilities, such as self-control, that will allow them to successfully accomplish the reasonable/desirable actions learned in class.

Furthermore, given that peers represent a model to draw inspiration from and conform to, carrying out social actions activates a virtuous circle of behaviors, self-efficacy, and self-esteem that reinforces one’s social identity and social role within the community [98].

Social campaigns to promote environmentally responsible behaviors help people engage in social actions from which they themselves or others will benefit. Individual actions (such as saving water or recycling) can become collective actions that enable people to improve the place in which they live and, consequently, to appreciate this place and perceive it as their own place of belonging.

The present study has some limitations that must be taken into consideration. First, the study was developed during the COVID-19 pandemic, so we had to use a convenience sample, which limited generalizability to the broader population of adolescents. Furthermore, we did not examine different ethnicities. The replication of the study with other samples is clearly required in order to ascertain the generalizability of the present findings and whether responses to pro-environmental behaviors vary depending on one’s ethnic background. Second, although the study was carried out through an anonymous online survey, social pressure may have encouraged desirable responses. Third, the cross-sectional nature of the study design precludes us from clearly determining the direction of the associations among the study variables. Thus, it would be important to conduct future studies in order to draw clearer conclusions about causality. Moreover, in the future it will be interesting to investigate other variables that may affect the implementation of pro-environmental behaviors, such as diet, life quality and lifestyle, motivations to apply this kind of behavior, civic engagement, and sense of community, as this could help us to better understand adolescents’ development.

Despite these limitations, the present study represents an attempt to analyze an important topic in the life of people, especially adolescents.

Moreover, in line with Bronfenbrenner’s ecological model [99], our findings suggest that adolescents’ pro-environmental behaviors may be associated with and affected by their social context—for example, parents or peers perceived as models. For this reason, it is important to make adolescents aware of the importance of natural resources [100]. Our results suggest that it would be useful to design educational programs to promote well-being by taking into consideration the role of parents and peers in influencing young people’s behaviors and emotions.

## Figures and Tables

**Table 1:** Descriptive statistics and Pearson’s *r* correlations.

	*M*	*SD*	Skewness	Kurtosis	1	2	3	4	5	6
1. Pro-environmental behavior	3.50	0.65	−0.48	1.11	-					
2. Place attachment	3.09	1.04	0.12	−0.33	0.12 **	-				
3. Personal well-being	3.36	0.73	−0.52	0.66	0.21 **	0.28 **	-			
4. Social well-being	2.75	1.04	0.50	0.06	0.21 **	0.24 **	0.48 **	-		
5. Gender	-	-	-	-	0.04	−0.08 **	−0.20 **	−0.10 **	-	
6. Age	16.26	1.46	−0.10	−0.10	−0.04	−0.11 **	−0.07 **	−0.11 **	−0.01	-

Note: Gender (1 = male, 2 = female) is a point-serial correlation. ** *p* < 0.01.

## Data Availability

The data presented in this study are available upon request from the corresponding author.
